# The Role of Immunohistochemical Markers in the Diagnosis of Early Myocardial Infarction

**DOI:** 10.7759/cureus.22391

**Published:** 2022-02-19

**Authors:** Aytül Buğra, Taner Daş

**Affiliations:** 1 Morgue Department, Histopathology Unit, The Council of Forensic Medicine, Istanbul, TUR

**Keywords:** immunohistochemistry, desmin, cathepsin s, troponin t, myoglobulin, cd59, fibronectin, myocardial infarction

## Abstract

Introduction: Pathological diagnosis of acute myocardial infarction can be difficult if death from ischemic injury has occurred within a short period of time. In this study, we aimed to determine the role of immunohistochemical markers in the diagnosis of early myocardial infarction.

Methods: The myocardium samples of 20 cases whose autopsies were performed at the Morgue Department of the Council of Forensic Medicine were evaluated. Hematoxylin and Eosin (H&E) stained slides and fibronectin, CD59, myoglobulin, troponin T, desmin, cathepsin S stained slides of 20 cases diagnosed with early myocardial infarction were retrospectively re-examined. The diagnosis of myocardial infarction was analyzed in two groups: Group 1: first eight hours, Group 2: 8-24 hours. The immunohistochemical staining patterns in these two groups were compared.

Results: Of the cases, 55% (n=11) had myocardial infarction consistent with the first eight hours, 45% (n=9) 8-24 hours with light microscopic examination. With fibronectin, 50% (n=10) of the cases showed Grade 1 staining, 5% (n=1) Grade 2, 15% (n=6) Grade 3 staining. The slides of three cases could not be reached. With CD59, 10% (n=2) of the cases showed Grade 1, 10% (n=2) Grade 2, 80% (n=16) Grade 3 staining. With troponin T, 50% (n=10) of the cases showed Grade 1, 45% (n=9) Grade 2, 5% (n=1) Grade 3 depletion. With cathepsin S, 10% (n=2) of the cases showed Grade 1 and 80% (n=16) Grade 3 depletion. The slides of two cases could not be reached. With desmin, 75% (n=15) had Grade 1 and 25% (n=5) Grade 2 depletion. Grade 3 depletion with myoglobulin was observed in all cases.

Conclusion: The diagnosis of early myocardial infarction, which may pose a problem for the forensic pathologist, may become easier with immunohistochemical methods. In cases where morphological findings are insufficient, it is more useful for diagnosis to be applied as a panel.

## Introduction

Myocardial infarction, which is the most common cause of cardiovascular death worldwide, occurs with ischemic stress caused by coronary artery occlusion and cardiomyocyte death [1]. Pathological diagnosis of acute myocardial infarction can be difficult if death from ischemic injury has occurred within a short period of time [2]. Wavy fibers, hypereosinophilia, nuclear changes, which are among the earliest microscopic changes, may not be immediately noticeable. The most obvious findings occur when neutrophils come to the necrotic environment, which is in the range of 6-12 hours at the earliest [3-5]. The emergence of these changes may be affected by many factors such as collateral flow, susceptibility of cardiomyocytes to ischemia [6]. Some catastrophic conditions, such as cardiac rupture, can cause a high and rapid death. In our previous study, we showed that myocardial rupture occurs mostly in the first 24 hours of myocardial infarction and that is followed by the third and seventh day of myocardial infarction as two peaks [7].

Recently, studies have been conducted to investigate the immunohistochemical methods that try to detect the molecules that accumulate in the cells or leak from the cells during ischemia with antibodies in order to show the damage to the cellular structures due to ischemia [2].

We thought that assisted staining methods would be useful for forensic pathologists to support the diagnosis in cases where histopathological findings for myocardial infarction were morphologically inadequate. With this idea, we aimed to determine the role of immunohistochemical markers in the diagnosis of myocardial infarction by comparing the results of immunohistochemical staining.

## Materials and methods

This study was carried out with the approval of the Education and Scientific Research Commission of the Council of Forensic Medicine (21589509/2018/968-25.12.2018). The myocardium samples of 20 cases whose autopsies were performed at the Morgue Department of the Council of Forensic Medicine were evaluated. Hematoxylin and Eosin (H&E) stained slides and fibronectin, CD59, myoglobulin, troponin T, desmin, cathepsin S stained slides of 20 cases diagnosed with early myocardial infarction were retrospectively re-examined. The diagnosis of myocardial infarction was analyzed in two groups: Group 1, first 8 hours, Group 2, 8-24 hours. The immunohistochemical staining patterns in these two groups were compared.

Histomorphological evaluation

H&E stained sections were examined under a light microscope. The cases were divided into two groups. Cases with morphological findings consistent with the first eight hours of myocardial ischemia (wavy appearance in muscle fibers, contraction band necrosis, nucleus loss, and presence of hypereosinophilia) were included in Group 1. Cases with neutrophil infiltration in addition to these findings were included in Group 2.

Immunohistochemical staining

BenchMark ULTRA, Roche’s fully automatic immunohistochemical slide staining system (Roche Holding AG, Basel, Switzerland) was used for immunohistochemical procedures. Immunohistochemical stainings with CD59 (Monoclonal Mouse antibody, dilution 1/100, Santa Cruz Biotechnology, Inc., Dallas, Texas, United States), fibronectin (Monoclonal Mouse antibody, dilution 1/200, Santa Cruz Biotechnology, Inc., Dallas, Texas, United States), cathepsin S (Santa Cruz Biotechnology, Inc., Dallas, Texas, United States), desmin (EnVision FLEX - Dako Omnis, Monoclonal Mouse Anti-Human Desmin, Clone D33, Ready to use, Agilent Technologies, Inc, Santa Clara, California, United States), troponin T, cardiac muscle (Rabbit Monoclonal Anti-Human Troponin T, Cardiac muscle, (TNNT2), Clone EP108, dilution 1/100, Epitomics, Inc., Burlingame, California, United States), Myoglobulin (Polyclonal Antibody to Human Myoglobulin, Ready to use, ScyTek Laboratories, Inc., Utah, United States) were applied to myocardial tissues for further evaluation of ischemic changes.

Since the immunohistochemical staining patterns were fibronectin deposition in the infarct area and CD59 deposition around the infarct area, and loss of staining for myoglobulin, desmin, troponin T, and cathepsin S in the infarct area, the findings were noted as staining for fibronectin and CD59, and depletion for myoglobulin, troponin T, desmin, cathepsin S.

Staining patterns were graded as Grade 1: detectable at highest magnification field (x400) with scanty fibers, Grade 2: detectable in patches at lower magnification fields (x200, x100), and Grade 3: detectable in larger areas at the lowest magnification field (x40). Staining patterns were evaluated using the intact myocardial tissue present in all slides as an internal control.

Statistical analysis

IBM SPSS Statistics for Windows, Version 21.0 (Released 2012. IBM Corp., Armonk, New York) was used for statistical analysis. While evaluating the study data, descriptive statistical methods (Average, Standard Deviation, Median, Frequency, Ratio, Minimum, Maximum), as well as the Kruskal-Wallis test, were used to compare qualitative data. Significance was evaluated at the p <0.05 level.

## Results

**Figure 1:**
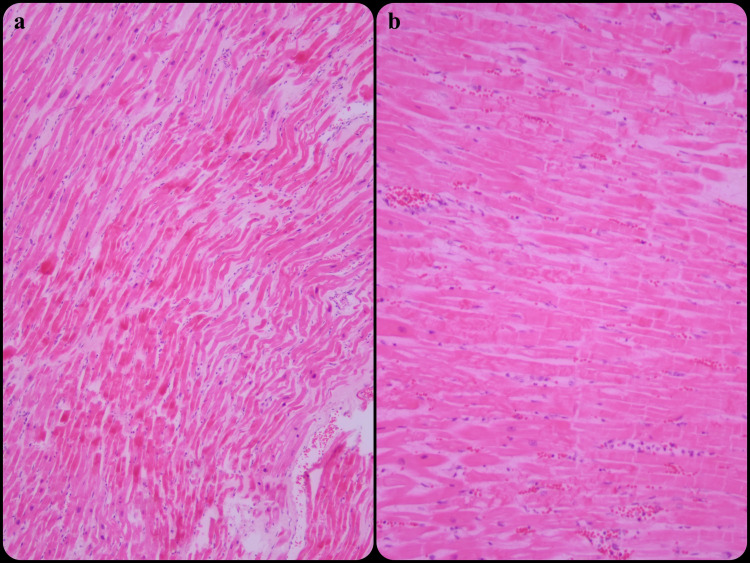
(a) Myocardial infarction consistent with the first eight hours (H&E, x100), (b) Myocardial infarction consistent with 8-24 hours (H&E, x100) H&E: hematoxylin and eosin staining

In this study, H&E stained slides and fibronectin, CD59, cathepsin S, troponin T, desmin, and myoglobulin stained immunohistochemistry slides of 20 cases diagnosed with early myocardial infarction were retrospectively analyzed. Of the cases, 55% (n=11) had myocardial infarction consistent with the first eight hours (Group 1), 45% (n=9) 8-24 hours (Group 2) with the light microscopic examination (Figure 1).

**Figure 2:**
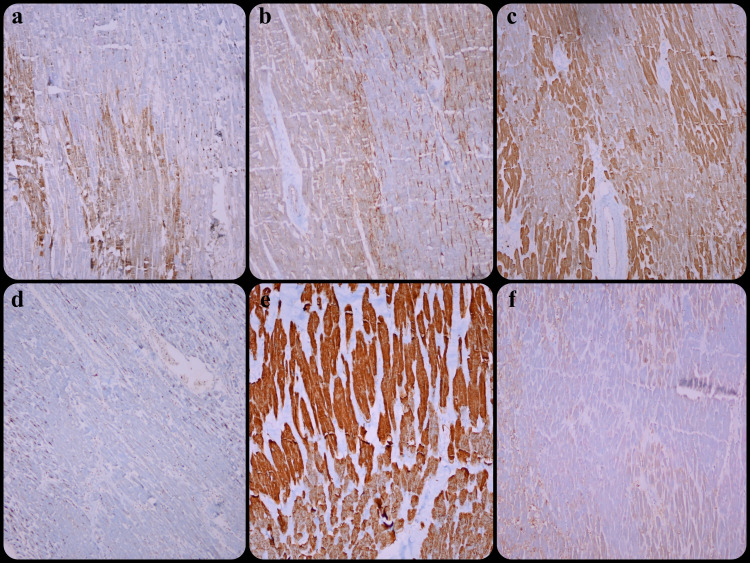
(a) Fibronectin staining (x100), (b) Staining of CD59 around necrosis (x100), (c) Depletion of troponin T (x100), (d) Depletion of cathepsin S (x100), (e) Depletion of desmin (x200), (e) Depletion of myoglobulin (x100)

With fibronectin, 50% (n=10) of the cases showed Grade 1 staining, 5% (n=1) Grade 2, and 15% (n=6) Grade 3 staining. The slides of three cases could not be reached. With CD59, 10% (n=2) of the cases showed Grade 1, 10% (n=2) Grade 2, and 80% (n=16) Grade 3 staining. With troponin T, 50% (n=10) of the cases showed Grade 1, 45% (n=9) Grade 2, and 5% (n=1) Grade 3 depletion. With cathepsin S, 10% (n=2) of the cases showed Grade 1 and 80% (n=16) Grade 3 depletion. The slides of two cases could not be reached. With desmin, 75% (n=15) had Grade 1 and 25% (n=5) Grade 2 depletion. Grade 3 depletion with myoglobulin was observed in all cases (Figure 2).

When the staining patterns of the immunohistochemical stains in both groups were compared, a significant depletion with troponin T and also significant staining with fibronectin was observed in Group 1. In the other immunohistochemical stains, there was no statistically significant difference between the two groups in terms of staining pattern (p>0.05). Data on the staining patterns of the cases are summarized in Table 1.

**Table 1:** Staining grades of immunohistochemical stains according to myocardial infarction time ^a^p value's calculated by Kruskal-Wallis test *Significantly different values

Immunohistochemical staining	Myocardial infarction time			p^a^ value
	Group 1 (first 8 hours) (n)	Group 2 (8-24 hours) (n)	Total (n/%)	
Fibronectin				0.013*
Grade 1	8	2	10 (50%)	
Grade 2	0	1	1 (5%)	
Grade 3	1	5	6 (15%)	
CD59				0.05
Grade 1	2	0	2 (10%)	
Grade 2	2	0	2 (10%)	
Grade 3	7	9	16 (80%)	
Troponin T				0.002*
Grade 1	9	1	10 (50%)	
Grade 2	2	7	9 (45%)	
Grade 3	0	1	1 (5%)	
Cathepsin S				0.145
Grade 1	2	0	2 (10%)	
Grade 2	0	0	0 (0%)	
Grade 3	7	9	16 (80%)	
Desmin				0.8
Grade 1	8	7	15 (75%)	
Grade 2	3	2	5 (25%)	
Grade 3	0	0	0 (0%)	
Myoglobulin				1
Grade 1	0	0	0 (0%)	
Grade 2	0	0	0 (0%)	
Grade 3	11	9	20 (100%)	

## Discussion

Morphological recognition of early myocardial infarction in forensic pathology may be difficult in cases where the findings are insufficient. The presence of morphologically uncertain findings, especially in terms of distinguishing between natural and unnatural deaths, may complicate the situation in cases where clinical, toxicological, and laboratory findings are insufficient. Autolytic changes that occur in the tissues during the postmortem period can also complicate the diagnosis.

For cardiomyocyte death to occur, a certain period of time must pass after the onset of ischemia. Although this period varies according to the sensitivity of the cardiomyocyte to necrosis, it may take at least six hours to reach the level where morphological changes can be detected both by macroscopic and microscopic [8]. Previously, some histochemical staining techniques have been used to detect early myocardial infarction [9-11]. Since these methods due to postmortem changes are not reliable, the use of these methods in the routine of forensic pathology is not very convenient [8]. It has been shown that immunohistochemical methods are more suitable for forensic pathology practice in detecting early myocardial infarction [12]. Studies have also shown that this method supports the diagnosis of myocardial damage [2,8,12]. We also showed early myocardial infarction with immunohistochemical stains in our study.

Studies have been conducted in which immunohistochemical markers applied as panels on various cellular components of the cardiomyocyte instead of a single marker were studied [2,8]. Molecules accumulating in the cardiomyocyte after an injury have also been investigated [2,13]. In our study, we investigated fibronectin and CD59, which accumulate in the cell with ischemic damage, and troponin T, cathepsin S, myoglobulin, and desmin, which are decreased in the cells.

Fibronectin accumulates in the cardiomyocyte after ischemic damage to the cell membrane. It is thought that it accumulates in plasma form due to disruption of plasma membrane integrity during myocardial infarction [14]. In a study, it was shown that fibronectin started to accumulate at the earliest one hour after ischemia [2]. In another study by Campobasso et al., it was shown that fibronectin accumulates 8-10 hours after the onset of cardiac symptoms; in this study, significant staining was shown in cases with long-term survival [8]. In our study, more intense and extensive staining with fibronectin, which was statistically significant, was observed in cases compatible with 8-24 hours of myocardial infarction. Our findings were also compatible with the study of Campobasso et al. We observed more extensive and more intense staining with longer survival time after myocardial ischemic injury.

The contribution of the activation of the complement system to cardiomyocyte damage has been investigated in many studies [15]. Studies have shown that with complement-mediated damage and ischemic injury, CD59 loss in the cell membrane. It has been shown that there is an increase in blood CD59 levels in acute myocardial infarction [16,17]. In our study, we showed that CD59 was depleted in the infarct area and accumulated around the necrotic area more than in other intact cardiomyocytes. We did not evaluate the blood level of CD59 in our study. More data may be revealed with studies to be carried out in larger series of immunohistochemical studies.

Troponin, which regulates the actin-myosin contractile complex, exists in muscle fibers in three isoforms: T, C, and I [8]. It has been shown in studies that troponin T is released from the cardiomyocytes in myocardial infarction and there is a loss of staining in necrotic areas [17]. In our study, we also found varying degrees of staining loss in necrotic areas in all cases in accordance with the literature.

Cathepsin S, a cysteine ​​protease localized in lysosomes, is expressed in cardiomyocytes [18]. In vitro studies showed an increase in cathepsin S expression in cardiomyocytes one to three days after myocardial infarction [1]. In our study, on the contrary, we observed depletion of cathepsin S in necrotic areas. In another study, cathepsin S level was found to be high in blood in myocardial infarction [19]. Cathepsin S was thought to be released from the necrotic area. We also thought that the loss of staining in the necrotic area in our study is due to the extracellular release of cathepsin S after necrosis. Studies examining cathepsin S in human myocardium are very limited in the literature. For this reason, new and comprehensive studies are needed.

Desmin and myoglobulin are also proteins that leak from cardiomyocytes with ischemic damage. Studies have shown that the sensitivity of myoglobulin is high [17]. In our study, a widespread and significant loss was observed with myoglobulin in all cases. Varying degrees of staining loss was observed with desmin. Our findings were found to be compatible with the literature.

Limitations

This study has some limitations. It is a retrospective study and some preparations could not be reached. Microscopic findings were re-evaluated. The absence of macroscopic findings is also a limitation of our study. Three slides of fibronectin and two slides of cathepsin S could not be reached. Since all samples are obtained in autopsies, there are varying grades of autolysis in all tissues due to the postmortem interval time. This may have affected the immunohistochemical staining patterns.

## Conclusions

The diagnosis of early myocardial infarction, which may pose a problem for the forensic pathologist, may become easier with immunohistochemical methods. In cases where morphological findings are insufficient, it is more useful for diagnosis to be applied as a panel. The results obtained from future studies may reveal markers that are useful for the diagnosis of early myocardial infarction.
